# Tensile performance modeling and process optimization of AA6061-T6/WC surface nanocomposites developed via friction stir processing

**DOI:** 10.1038/s41598-026-49260-1

**Published:** 2026-04-30

**Authors:** Saleh S. Abdelhady, Rehab E. Elbadawi

**Affiliations:** 1 Department of Mechanical Engineering, Higher Technological Institute, P.O. Box: 228, 10th of Ramadan City, Egypt; 2 Department of Mechanical Engineering, Mechatronics Engineering Program, Egyptian Academy for Engineering and Advanced Technology (EAEAT), Cairo City, Egypt

**Keywords:** WC nanoparticle, Friction stir processing, Stir zone, Tensile properties, Engineering, Materials science, Nanoscience and technology

## Abstract

In the present investigation, friction stir processing (FSP) is implemented to develop the tungsten carbide (WC) nanoparticle-reinforced aluminum alloy Al6061-T6. The tensile properties of the Al6061-T6/WC surface nanocomposite were evaluated in relation to the volume fraction of WC nanoparticles, the number of passes, rotation speed, and traverse speed. The experiments were designed using the Box-Behnken Design (BBD) of response surface methodology (RSM). For identifying the significant variables and interaction implications, analysis of variance (ANOVA) was performed. The models generated demonstrate that rotating speed is the most significant variable and transverse speed is of little importance. Heat input to FSP increases as traverse speed decreases and tool rotational speed increases. Increasing the number of FSP passes effectively broke the coarse and dendritic clusters, refined the matrix grains, and dynamic recrystallization (DRX) resulting in equiaxed grains that, through restricted dislocation activity, exhibit tensile behavior. Furthermore, the extreme plastic deformation and heat production during FSP results in the breakage of WC particles and coarse particles, the removal of porous holes, and DRX of an ultrafine grain-sized structure. The optimized surface nanocomposite with the highest tensile strength (315 MPa), yield strength (221 MPa), and elongation (9.7%) was achieved at volume fraction 2%, number of passes 5, rotation speed 1000 rpm, and traverse speed 30 mm/min. The surface composite that developed has been identified as an appropriate material for the automotive, aerospace, marine, defense, and transportation sectors, among others, that require lightweight and improved surface qualities.

## Introduction

Metal matrix composites (MMCs) comprised of metallic matrices reinforced with ceramic or metallic materials to provide superior stiffness, strength, thermal stability, and wear resistance compared to monolithic metals^1,2^. Aluminum is used as the matrix in aluminum matrix composites (AMCs) due to its low density, resistance to corrosion, and ease of manufacturing; when reinforced with particulates, whiskers, or nanoparticles, this allows for increased specific strength and fatigue resistance^3,4^. Strengthening mechanisms involving load transfer, Orowan looping, interfacial constraint, and grain refinement control the composite response^5^. Based on interfacial bonding and dislocations caused by thermal mismatch, carbide, nitride, and oxide material reinforcement exhibit unique hardening pathways^6^. AMCs are used in aerospace structures, automotive powertrain components, thermal management devices, and defense systems that require lightweight and high-performance materials^7^. Their operational capacity in pistons, brake systems, valves, and wear-intensive mechanical components supports the replacement of conventionally alloyed aluminum when combined strength-wear requirements exceed monolithic limits^8^.

AA6061-T6 is a heat-treatable aluminum alloy tempered by the precipitation of Mg_2_Si phases, offering a combination of high strength, corrosion resistance, and superior machinability^9,10^. The T6 temper involves solution heat treatment followed by artificial aging, producing a microstructure developed for mechanical performance^11^. AA6061-T6 is utilized extensively in aerospace structures, marine frameworks, automotive components, and bicycle frames due to its superior strength-to-weight ratio and durability^12,13^. Its weldability generally is good, and friction stir welding or processing can further improve localized mechanical characteristics, refine grain structure, and improve fatigue resistance^14,15^. As a matrix in aluminum composites, AA6061-T6 integrates effectively with ceramic additives such as SiC, Al_2_O_3_, and TiC, improving wear resistance and stiffness^16,17^. Processing methods including stir casting, powder metallurgy, and friction stir processing ensure uniform reinforcement distribution and enhance mechanical behavior^18,19^. Furthermore, to its mechanical strength, AA6061-T6 has good weldability and formability, which allows it to be processed using advanced solid-state techniques such as friction stir welding and friction stir processing to further enhance mechanical performance and microstructural refinement^20–22^. The AA6061 aluminum alloy’s mechanical strength, wear resistance, and microstructural properties are all significantly improved by the addition of ceramic particles^23^.

Friction stir processing (FSP) modifies microstructures through severe plastic deformation excluding melting, generating tiny equiaxed grains through dynamic recrystallization^24^. Frictional heating eliminates casting flaws; dissolves segregated phases and homogenizes solute distributions by softening the material and creating intense plastic flow^25^. High strain rates and regulated thermal cycles lead to grain refinement to extremely small and nanocrystalline scales^26^. Heat input and mixing efficiency are determined by procedure variables such as rotation speed, traverse speed, plunge depth, and shoulder geometry^27^. Aluminum alloys respond successfully since the controlled temperature distribution avoids overheating while promoting dynamic recrystallization^28^. FSP permits the introduction of ceramic nanoparticles, oxides, carbides, and carbonaceous additives through solid-state dispersion, avoiding interfacial reactions common in liquid processing^29^. The absence of melting avoids the formation of brittle intermetallics, establishing high-quality interfaces^30^. The base material has a high density of needle-shaped precipitates; either the precipitates undergo strain-induced dissolution at high temperatures during processing, or their quantity decreases following FSP^31^. The approach is appreciated for its repeatability, scalability, and particular microstructural engineering^32^.

Nanoparticles such as Al_2_O_3_, SiC, TiC, TiO_2_, B_4_C, carbon nano tubes CNTs, graphene, ZrO_2_, and hexagonal boron nitride (h-BN) enhance fatigue performance, toughness, wear resistance, and thermal stability in aluminum alloys^33^. They strengthen load transfer, restrict dislocation motion, and refine grains through high interface area^34^. While carbides like SiC, WC, TiC, and B_4_C enhance hardness and abrasion resistance, oxide reinforcements like Al_2_O_3_ and ZrO_2_ contribute to stability at high temperatures^35^. Carbon-based reinforcements (CNTs, graphene) enhance stiffness while reducing friction via solid-lubricating behavior^36^. Hybrid nanocomposites incorporate various reinforcement mechanisms for synergistic efficiency^37^. Processing methods—including FSP, mechanical alloying, ultrasonic-assisted casting, and spark plasma sintering—reduce aggregates and maintain stable interfaces^38^. Development of surface Al matrix composites by adding ceramics to form a particle across the plunger. Additional tests of FSP surface composites have shown improvements in tribological properties, especially in surfaces based on aluminum^39^. The choosing of reinforced materials relies on functional requirements: SiC for wear-intensive parts, TiC for cutting tools, graphene for conductivity, and oxides for thermal stability^40^.

Tungsten carbide (WC) nanoparticles contribute excellent hardness, stiffness, and thermal resilience to aluminum matrices, exhibiting superior wear resistance and mechanical strength^41^. At nanoscale dimensions, WC promotes grain refinement via Zener pinning and restricts dislocation movement, enhancing hardness and strength^42^. Liquid-state dispersion of WC is challenging owing to sedimentation and interfacial reactions^43^. Under extreme plastic deformation, solid-state techniques like FSP, mechanical alloying, and hot extrusion produce consistent WC integration^44^. The incorporation of nano-WC particles caused the microstructure to change from columnar to equiaxed, which greatly increased the tensile strength^45^. WC nanoparticles enhanced crack deflection, abrasion resistance, and extreme-temperature dimensional stability^46^. They have applications in cutting tools, brake elements, sliding interfaces, and erosion-resistant surfaces^47^. WC uniformly disperses during FSP, resulting in stable interfaces and refined grains^48^. The wear resistance of particle-reinforced aluminum alloys was found to be influenced by the size, shape, type, and volume of the particles. Using WC and other hard particles as reinforcement particles has increased the wear resistance of friction stir welded joints of non-heat treatable aluminum alloys^49,50^.

FSP optimization requires quantifying the effects of rotation speed, traverse speed, tool geometry, plunge depth, and reinforcement concentration on mechanical performance^51^. By modeling nonlinear behavior, Response Surface Methodology (RSM) generates predictive equations for particle dispersion, defect suppression, and microstructural refinement^52^. Central Composite Design and Box–Behnken Design reduce experimental runs while exhibiting curvature effects in response surfaces^53^. Grain size, dislocation density, and reinforcement distribution are correlated with mechanical results using RSM^54^. Tensile strength, wear resistance, and hardness can all be improved simultaneously through multi-objective optimization with desirability functions^55^. RSM modeling indicates optimal processing conditions for aluminum nanocomposites fabricated via FSP^56^. This method of statistics eliminates trial-and-error, providing scalable and reproducible FSP effectiveness across alloy frames^57^.

Recently, scientists have successfully added metallic particles to MMCs as reinforcement, increasing their strength and ductility retention. Even though a lot of work has been done, more investigation is still required to determine how to describe the addition of WC nanoparticles in the AA 6061-T6 matrix using the FSP technique. Furthermore, a significant amount of research has been focused on FSP single objective problem optimization of process parameters, according to the literature review. Research on the optimization of process parameters by considering multiple mechanical characteristics is rare. Therefore, an attempt has been made in this investigation to optimize a few significant FSP parameters to maximize the tensile performance in the friction stir processing AA6061-T6 nanocomposite using the Box-Behnken Design of response surface methodology. An empirical relationship was established between the input parameters (volume fraction, number of passes, tool rotational speed, and traverse speed) and the output responses (tensile strength, yield strength, and percentage elongation of WC nanoparticles-based friction stir processing AA6061-T6 nanocomposite) to identify the optimal FSP processing parameters.

## Experimental Procedure

### Materials

**Fig. 1:**
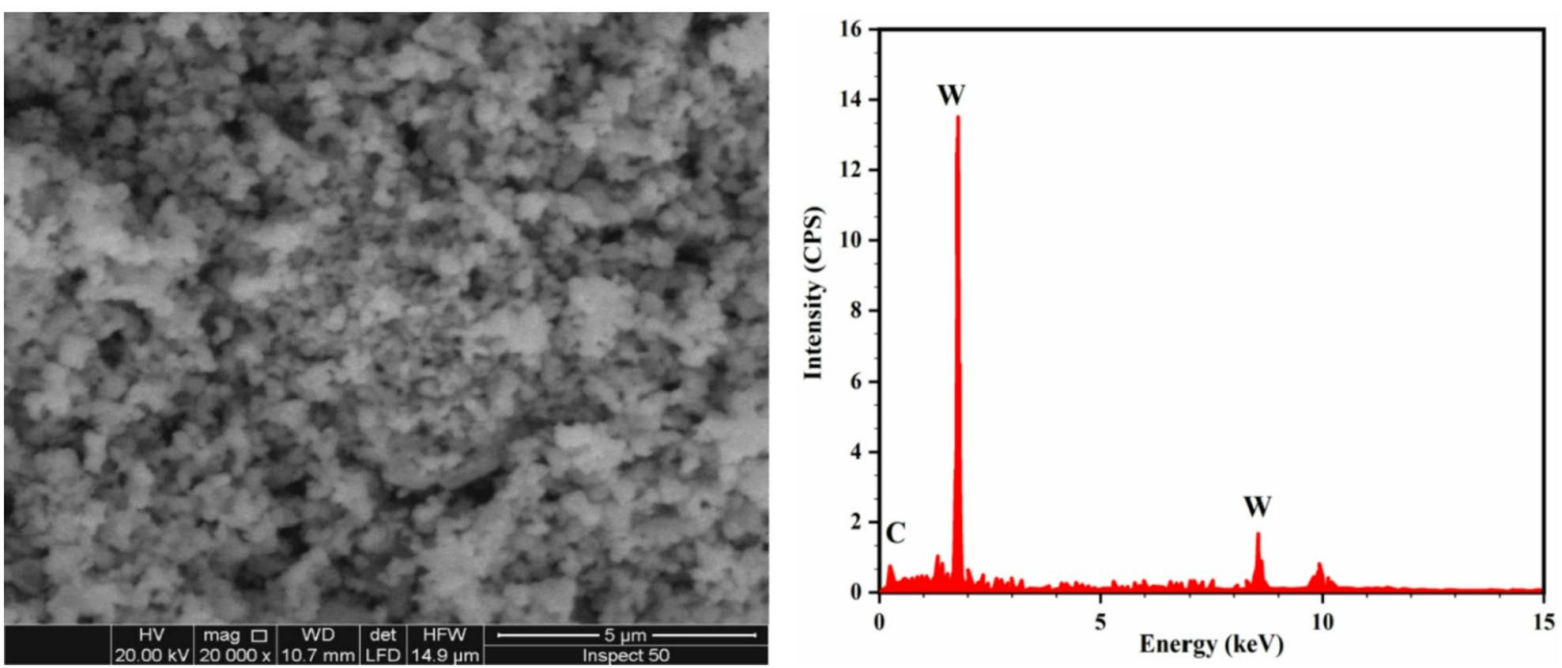
SEM micrograph and EDX of the as-received WC reinforcing particulates.

The as-received AA6061-T6 used as the base material have the chemical composition displayed in Table 1. Tungsten carbide (WC) nanoparticles were supplied by Sigma-Aldrich with size range of < 200 nm were utilized as the reinforcement. The size and shape of the reinforcing nanoparticles as determined by scanning electron microscopy (SEM) are shown in Fig. 1.

**Table 1:** Chemical composition (wt%) of AA6061-T6 sheet.

Element	Mg	Fe	Si	Cu	Cr	Al.
Wt.%	1.09	0.12	0.19	0.47	0.45	Bal.

### Friction stir processes (FSP)

**Fig. 2:**
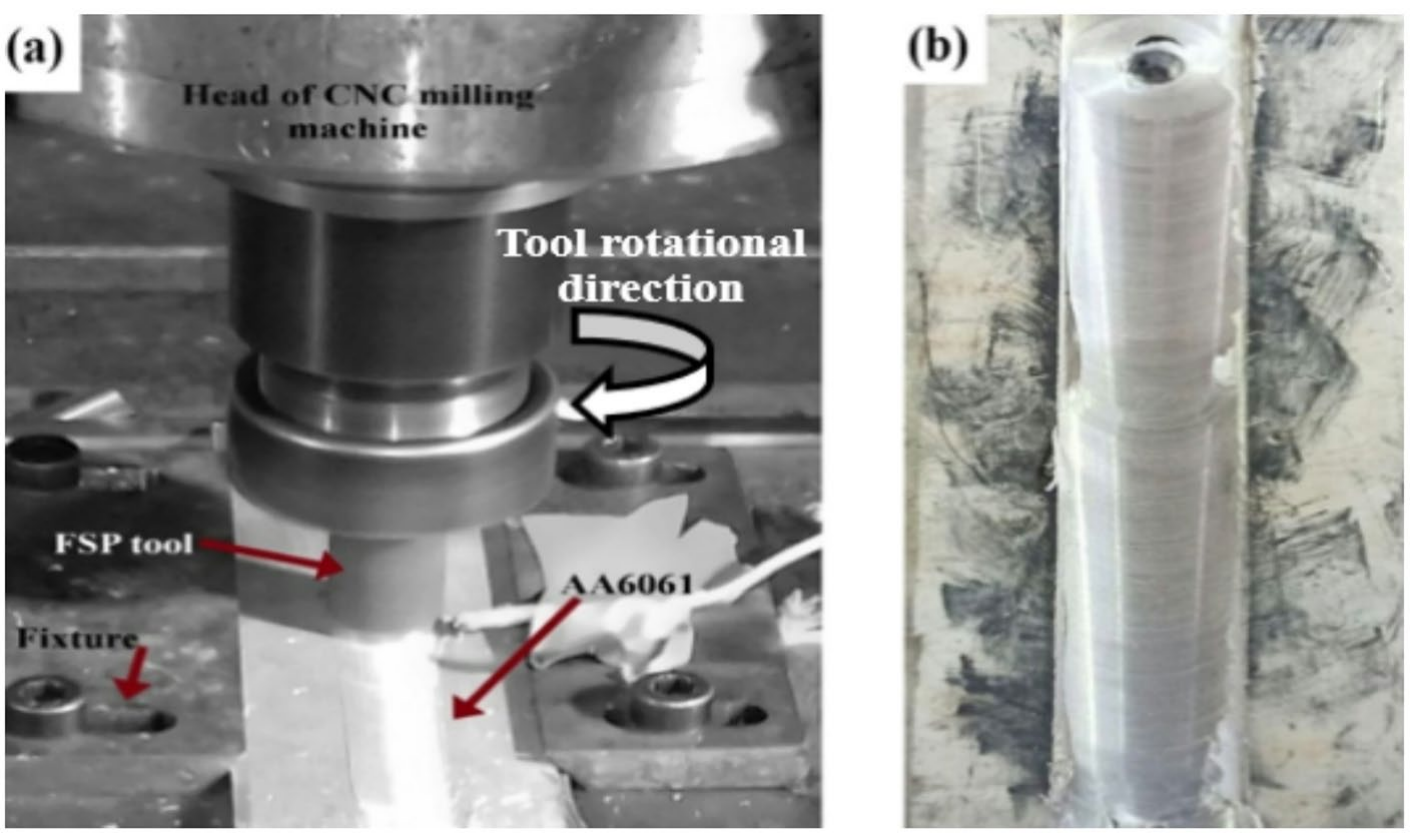
(**a**) Macrograph of FSP procedure and (**b**) appearance of FSP Al6061 with WC nano particles.

**Fig. 3:**
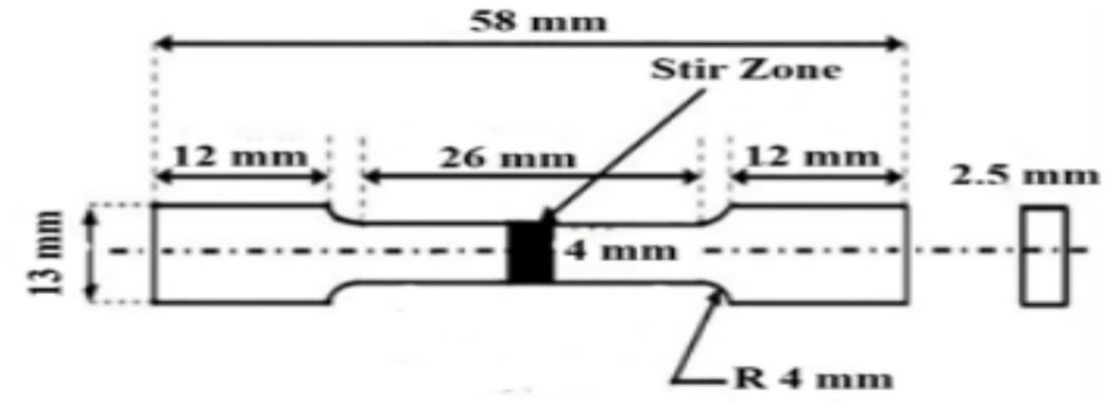
Dimensions of flat tensile specimen.

Aluminum alloy sheets (AA6061-T6) were wire-cut with dimensions of 150 mm× 50 mm ×6 mm. FSP was conducted using a computer numerical control (CNC) milling machine. Rectangular grooves were machined with varying depth levels to create different volume fractions (V_f_) of WC particles. The volume of each reinforcing particle was calculated using Eq. 1^58,59^. The groove dimensions of 150 × 1 × 0.6 mm (length × width × depth) for 2% V_f_ and 150 × 1 × 1.2 mm for 4% V_f_. The specimens were set firmly on the CNC milling machine using a special fixture as shown in Fig. 3. After creating the groove in the middle of the plate, filling it with WC particles, and sealing it using a pin-less tool to prevent Particles from escaping during the FSP process. The reinforcement particles were inserted into the matrix using a hardened H13 steel tool which had an M6 threaded pin profile, a 5 mm height, and an 18 mm shoulder diameter. A wire cut electrical discharge machine (EDM) was used to cut FSP specimens, both with and without WC particles, from the middle of the pass in a direction perpendicular to the processing method to perform a mechanical and microstructural examination (Fig. 2).

1 $${\mathrm{Volume}}\:{\mathrm{fraction}} = \frac{{{\mathrm{depth}}\:{\mathrm{of}}\:{\mathrm{groove}}\: \times \:{\mathrm{width}}\:{\mathrm{of}}\:{\mathrm{groove}}}}{{{\mathrm{diameter}}\:{\mathrm{of}}\:{\mathrm{pin}}\: \times \:{\mathrm{length}}\:{\mathrm{of}}\:{\mathrm{pin}}\:}}$$

### Box-Behnken design-based response surface methodology

Response Surface Methodology (RSM) has developed into an essential statistical and optimization tool in engineering research because it enables the methodical examination of complex process variables and their interactions to model and improve system performance with minimal investigation effort. Regression analysis, structured experimental designs, and mathematical modeling are all combined in RSM to fit polynomial equations, usually second-order models, that can accurately represent nonlinear behavior and curvature in process responses^60^. The Box-Behnken Design (BBD) is considered one of the most effective and practical RSM designs, particularly in materials and manufacturing studies where full factorial or central composite designs may be prohibitively expensive or involve extreme factor combinations that are challenging to physically implement. BBD is particularly well suited for FSP experiments because it avoids extreme corner points and requires fewer experimental runs than central composite designs^61^. In FSP-based surface composites, this work shows that BBD-based RSM accurately predicts the effects of reinforcement volume fraction (V_f_), number of passes, tool rotation speed (RS), and traverse speed (TS) on tensile strength, yield strength, and elongation. Table 2 displays the levels associated with each of the four input variables with a zero-degree tool tilt angle and a 0.2 mm shoulder plunge depth. Twenty-seven experimental runs were chosen by modeling the responses using a second order (quadratic) approach with four variables at three levels of BBD.

**Table 2:** The levels of the input variables used in BBD.

Variables	Units	Symbol	Levels		
			− 1	0	1
Volume fraction	(%)	X_1_	0	2	4
Number of passes		X_2_	1	3	5
Rotation speed	RPM	X_3_	800	1000	1200
Traverse speed	mm/min	X_4_	30	50	70

Design-Expert Software Version 12 was used to obtain the response surface model. The experimental design matrix and their coded values for different runs and variables are shown in Table 3. Equation 2, a second-order polynomial, represents the response surface model that can be suggested between each response y and the variables x.

2 $$\begin{aligned} y = & \;a_{0} + \sum\limits_{{i = 1}}^{n} {a_{i} X_{i} } + \sum\limits_{{i = 1}}^{n} {a_{{ii}} X_{i}^{2} } \\ & + \sum\limits_{{i = 1}}^{{n - 1}} {\sum\limits_{{j = i + 1}}^{n} {a_{{ij}} X_{i} X_{J} } } \\ \end{aligned}$$

where X_i_ and X_j_ are the coded values of the input variables, n is the number of factors, a_0_ is a constant coefficient, a_i_ is the regression coefficient for linear effects, a_ii_ is the quadratic coefficient, and a_ij_ is the interaction coefficient. The statistical significance of the provided mathematical models was assessed using statistical analysis of variance (ANOVA). The significance of each term in the developed model can be limited by examining F and P-values. P-values less than 0.05 indicate that the variable has a significant impact on the responses. The adjusted R^2^ and coefficient of determination (R^2^) were examined to confirm that the appropriate model is accurate and appropriate for the intended use. R^2^ indicates the proportion of the total variability that the regression model can account for. Higher values of (R^2^) and adjusted-R^2^ indicate the degree of significance to which the model fits the experimental data.

**Table 3:** Design matrix and different variable values (coded and actual) carried out using BBD and experimental results.

Exp. no.	Coded variables				Actual variables				Experimental responses		
	X_1_	X_2_	X_3_	X_4_	V_f_	No. of passes	RS	TS	Tensile strength	Yield strength	Elongation (%)
1	− 1	− 1	0	0	0	1	1000	50	198	139	9.9
2	1	− 1	0	0	4	1	1000	50	177	124	5.4
3	− 1	1	0	0	0	5	1000	50	235	165	8.3
4	1	1	0	0	4	5	1000	50	280	196	8.8
5	0	0	− 1	− 1	2	3	800	30	159	112	9.5
6	0	0	1	− 1	2	3	1200	30	225	158	6.6
7	0	0	− 1	1	2	3	800	70	145	102	7.4
8	0	0	1	1	2	3	1200	70	271	190	6.4
9	− 1	0	0	− 1	0	3	1000	30	167	117	8.9
10	1	0	0	− 1	4	3	1000	30	202	142	6.5
11	− 1	0	0	1	0	3	1000	70	195	137	7.4
12	1	0	0	1	4	3	1000	70	182	128	5.2
13	0	− 1	− 1	0	2	1	800	50	135	95	8
14	0	1	− 1	0	2	5	800	50	165	116	9.4
15	0	− 1	1	0	2	1	1200	50	238	167	7.6
16	0	1	1	0	2	5	1200	50	305	214	7.9
17	− 1	0	− 1	0	0	3	800	50	112	79	9
18	1	0	− 1	0	4	3	800	50	119	84	8.4
19	− 1	0	1	0	0	3	1200	50	235	165	8.6
20	1	0	1	0	4	3	1200	50	210	147	5.1
21	0	− 1	0	− 1	2	1	1000	30	202	142	7.8
22	0	1	0	− 1	2	5	1000	30	315	221	9.7
23	0	− 1	0	1	2	1	1000	70	255	179	8.3
24	0	1	0	1	2	5	1000	70	241	169	7
25	0	0	0	0	2	3	1000	50	291	204	9.4
26	0	0	0	0	2	3	1000	50	281	197	9.6
27	0	0	0	0	2	3	1000	50	295	207	9.1

### Microstructural evaluations

Microstructural assessments were carried out at the Tabbin Institute for Metallurgical Studies (TIMS) in Egypt by employing optical microscopy (Leco LX 31-USA) and SEM model Quanta 250FEG (Field Emission Gun) with EDX (Energy Dispersive X-ray Analyses) was used to analyze the distribution of reinforcement particles following FSP. Samples with dimensions of 20 mm x 10 mm x 6 mm were cut transversely at the friction stir processing cross section. The surfaces of all the specimens were polished and ground to a mirror-like sheen prior to displaying the surface morphology of each one. The specimens were polished mechanically, and then Keller’s reagent (190 mL distilled water, 5 mL nitric acid, 3 mL hydrochloric acid, and 2 mL hydrofluoric acid) was used to etch them.

### Mechanical characterization

The tensile specimen is developed using wire cut electrical discharge machine (EDM) in accordance with American Society for Testing and Materials (ASTM E8M-04 ) standards, with dimensions of 2.5 mm thick, 4 mm wide, 58 mm long, and a gauge length of 26 mm parallel to the direction of the composite as shown in Fig. 3.

## Results and discussion

### Developing equation-based mathematical models and adequate suitability for predicting responses

Based on response surface methodology, twenty-seven experiments were created using BBD for regression analysis of four variables at three levels (Table 3). The following shows the statistically determined tensile strength, yield strength, and elongation for each procedure trial. The mathematical model was established using the estimated coefficient values from Eq. 2. The mathematical expression for the best-fitting models in the data set based on coded variables is presented in Eqs. 3, 4, and 5.

3 $$\begin{aligned} {\mathrm{Y}}_{1} ({\mathrm{Tensile}}\;{\mathrm{strength}}) = & + 17.00 + 0.0783{\mathrm{X}}_{1} + 0.9202{\mathrm{X}}_{2} \\ & + 1.96{\mathrm{X}}_{3} + 0.0621{\mathrm{X}}_{4} - 2.09{\mathrm{X}}_{1}^{2} \\ & - 0.2296{\mathrm{X}}_{2}^{2} - 2.16{\mathrm{X}}_{3}^{2} - 0.9909{\mathrm{X}}_{4}^{2} \\ & + 0.5427{\mathrm{X}}_{1} {\mathrm{X}}_{2} - 0.2910{\mathrm{X}}_{1} {\mathrm{X}}_{3} - 0.4408{\mathrm{X}}_{1} {\mathrm{X}}_{4} \\ & + 0.2027{\mathrm{X}}_{2} {\mathrm{X}}_{3} - 0.9950{\mathrm{X}}_{2} {\mathrm{X}}_{4} + 0.5075{\mathrm{X}}_{3} {\mathrm{X}}_{4} \\ \end{aligned}$$

4 $$\begin{aligned} {\mathrm{Y}}_{2} ({\mathrm{Yield}}\;{\mathrm{strength}}) = & + 14.24 + 0.0638{\mathrm{X}}_{1} + 0.7683{\mathrm{X}}_{2} + 1.64{\mathrm{X}}_{3} \\ & + 0.0511{\mathrm{X}}_{4} - 1.75{\mathrm{X}}_{1}^{2} - 0.1930{\mathrm{X}}_{2}^{2} \\ & - 1.8{\mathrm{X}}_{3}^{2} - 0.8245{\mathrm{X}}_{4}^{2} + 0.4523{\mathrm{X}}_{1} {\mathrm{X}}_{2} \\ & - 0.2495{\mathrm{X}}_{1} {\mathrm{X}}_{3} - 0.3727{\mathrm{X}}_{1} {\mathrm{X}}_{4} + 0.1706{\mathrm{X}}_{2} {\mathrm{X}}_{3} \\ & - 0.8322{\mathrm{X}}_{2} {\mathrm{X}}_{4} + 0.4244{\mathrm{X}}_{3} {\mathrm{X}}_{4} \\ \end{aligned}$$

5 $$\begin{aligned} {\mathrm{Y}}_{3} ({\mathrm{Elongation}}) = & + 3.06 - 0.1989{\mathrm{X}}_{1} + 0.0629{\mathrm{X}}_{2} \\ & - 0.1448{\mathrm{X}}_{3} - 0.1095{\mathrm{X}}_{4} - 0.1934{\mathrm{X}}_{1}^{2} \\ & - 0.0342{\mathrm{X}}_{2}^{2} - 0.1293{\mathrm{X}}_{3}^{2} - 0.2023{\mathrm{X}}_{4}^{2} \\ & + 0.2270{\mathrm{X}}_{1} {\mathrm{X}}_{2} - 0.1431{\mathrm{X}}_{1} {\mathrm{X}}_{3} - 0.0015{\mathrm{X}}_{1} {\mathrm{X}}_{4} \\ & - 0.0459{\mathrm{X}}_{2} {\mathrm{X}}_{3} - 0.1392{\mathrm{X}}_{2} {\mathrm{X}}_{4} + 0.0807{\mathrm{X}}_{3} {\mathrm{X}}_{4} \\ \end{aligned}$$

where Y_1_, Y_2_, and Y_3_ exhibit the tensile strength (MPa), yield strength (MPa) and elongation (%), respectively, and X_1_, X_2_, X_3_and X_4_ are the volume fraction of WC, number of passes, rotation speed and traverse speed, respectively.

A reliable and popular statistical approach for assessing the importance, adequateness, and predictive power of developed models is analysis of variance (ANOVA), especially in experimental investigations with numerous input variables^62^. F-values and p-values are used to assess the statistical significance of the model and its terms; a p-value of less than 0.05 typically indicates that the corresponding factor has a statistically significant impact on the response. The reliability of the fitted model is confirmed by a high model F-value, which indicates that the regression equation accounts for a significant amount of the observed variation in comparison to random error^63,64^.

Tensile strength, yield strength, and elongation ANOVA results based on a simplified polynomial mode are displayed in Tables 4 and 5, and 6, respectively. The results of the ANOVA test showed that all three models had F-values and p-values less than 0.05, meaning that the developed models had a confidence level greater than 95% and could be used to investigate the design space^65^. F-values of 39.43, 39.15, and 23.15 for tensile strength, yield strength, and elongation, respectively, demonstrate the significance of the developed models. While the p-value is less than 0.05, the terms in the model are significant; when the p-value is greater than 0.05, they are not significant. The model terms X_2_, X_3_, X_1_ × _2_, X_2_ × _4_, X_3_ × _4_, X_1_^2^, X_3_^2^ and X_4_^2^ are significant for the tensile strength (p-value ≤ 0.05), according to Table 4, which displays the results of an optimization inquiry using ANOVA analysis. Also, NOVA results in Table 5 presented that the model terms X_2_, X_3_, X_1_ × _2_, X_2_ × _4_, X_3_ × _4_, X_1_^2^, X_3_^2^ and X_4_^2^ are significant for the yield strength (p-value ≤ 0.05). The model variables X_1_, X_2_, X_3_, X_4,_ X_1_ × _2_, X_1_ × _3_, X_2_ × _3_, X_3_ × _4,_ X_1_^2^, X_3_^2^_,_ and X_4_^2^ are significant for elongation (p-value ≤ 0.05), according to the NOVA results in Table 6.The R^2^ value of the model for tensile strength, yield strength, and elongation is 0.9787, 0.9786, and 0.9643, respectively. Furthermore, the adjusted R^2^ values are 0.9539,0.9536, and 0.9226. The results demonstrated the models’ substantial significance.

**Fig. 4:**
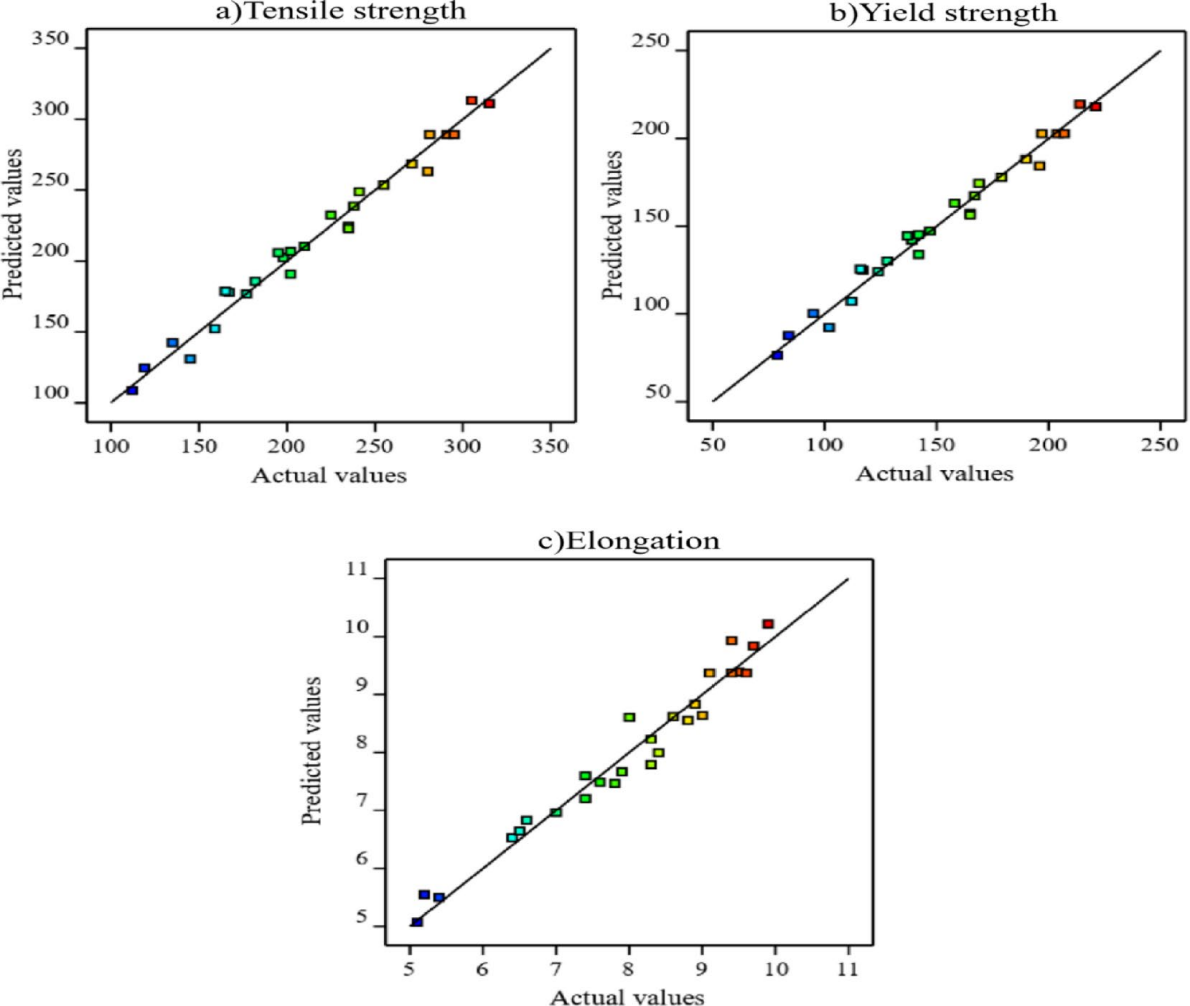
Predicted against actual values of (**a**) tensile strength, (**b**) yield strength and (**c**) elongation.

Figure 4 compares the tensile strength, yield strength, and elongation measured by experiments with the predictions made by the RSM models. A diagonal line that displays a random uniform scatter of all the points represented by a straight line is used to compare the predicted and actual results^66^. Tensile strength (Fig. 4a), yield strength (Fig. 4b), and elongation (Fig. 4c) all show a strong correlation between the experimental and predicted results, indicating the successful establishment of empirical correlations.

**Table 4:** Analysis of variance (ANOVA) of the response surface mathematical model for tensile strength.

Source	Sum of squares	DF	Mean square	F-value	*P*-value	
Model	104.77	14	7.48	39.43	< 0.0001	Significant
$\:{\mathrm{X}}_{1}$	0.0736	1	0.0736	0.3880	0.5450	
$\:{\mathrm{X}}_{2}$	10.16	1	10.16	53.54	< 0.0001	
$\:{\mathrm{X}}_{3}$	46.29	1	46.29	243.89	< 0.0001	
$\:{\mathrm{X}}_{4}$	0.0463	1	0.0463	0.2441	0.6302	
$\:{\mathrm{X}}_{1}{\mathrm{X}}_{2}$	1.18	1	1.18	6.21	0.0284	
$\:{\mathrm{X}}_{1}{\mathrm{X}}_{3}$	0.3387	1	0.3387	1.78	0.2063	
$\:{\mathrm{X}}_{1}{\mathrm{X}}_{4}$	0.7773	1	0.7773	4.10	0.0658	
$\:{\mathrm{X}}_{2}{\mathrm{X}}_{3}$	0.1643	1	0.1643	0.8658	0.3705	
$\:{\mathrm{X}}_{2}{\mathrm{X}}_{4}$	3.96	1	3.96	20.87	0.0006	
$\:{\mathrm{X}}_{3}{\mathrm{X}}_{4}$	1.03	1	1.03	5.43	0.0381	
$\:{\mathrm{X}}_{1}^{\:\:2}$	23.36	1	23.36	123.09	< 0.0001	
$\:{\mathrm{X}}_{2}^{\:\:2}$	0.2811	1	0.2811	1.48	0.2470	
$\:{\mathrm{X}}_{3}^{\:\:\:2}$	24.95	1	24.95	131.46	< 0.0001	
$\:{\mathrm{X}}_{4}^{\:\:\:2}$	5.24	1	5.24	27.59	0.0002	
Residual	2.28	12	0.1898			
Lack of Fit	2.19	10	0.2187	4.84	0.1834	Not significant
Pure Error	0.0904	2	0.0452			
Cor Total	107.05	26				

**Table 5:** Analysis of variance (ANOVA) of the response surface mathematical model for yield strength.

Source	Sum of squares	DF	Mean square	F-value	*P*-value	
Model	72.86	14	5.20	39.15	< 0.0001	Significant
$\:{\mathrm{X}}_{1}$	0.0488	1	0.0488	0.3672	0.5558	
$\:{\mathrm{X}}_{2}$	7.08	1	7.08	53.30	< 0.0001	
$\:{\mathrm{X}}_{3}$	32.09	1	32.09	241.39	< 0.0001	
$\:{\mathrm{X}}_{4}$	0.0313	1	0.0313	0.2354	0.6363	
$\:{\mathrm{X}}_{1}{\mathrm{X}}_{2}$	0.8182	1	0.8182	6.16	0.0289	
$\:{\mathrm{X}}_{1}{\mathrm{X}}_{3}$	0.2489	1	0.2489	1.87	0.1962	
$\:{\mathrm{X}}_{1}{\mathrm{X}}_{4}$	0.5556	1	0.5556	4.18	0.0635	
$\:{\mathrm{X}}_{2}{\mathrm{X}}_{3}$	0.1164	1	0.1164	0.8758	0.3678	
$\:{\mathrm{X}}_{2}{\mathrm{X}}_{4}$	2.77	1	2.77	20.84	0.0006	
$\:{\mathrm{X}}_{3}{\mathrm{X}}_{4}$	0.7206	1	0.7206	5.42	0.0382	
$\:{\mathrm{X}}_{1}^{\:\:2}$	16.33	1	16.33	122.89	< 0.0001	
$\:{\mathrm{X}}_{2}^{\:\:2}$	0.1988	1	0.1988	1.50	0.2449	
$\:{\mathrm{X}}_{3}^{\:\:\:2}$	17.32	1	17.32	130.32	< 0.0001	
$\:{\mathrm{X}}_{4}^{\:\:\:2}$	3.63	1	3.63	27.28	0.0002	
Residual	1.60	12	0.1329			
Lack of Fit	1.53	10	0.1530	4.69	0.1886	Not significant
Pure Error	0.0653	2	0.0326			
Cor Total	74.45	26				

**Table 6:** Analysis of variance (ANOVA) of the response surface mathematical model for elongation.

Source	Sum of squares	DF	Mean square	F-value	*P*-value	
Model	1.67	14	0.1193	23.15	< 0.0001	Significant
$\:{\mathrm{X}}_{1}$	0.4747	1	0.4747	92.10	< 0.0001	
$\:{\mathrm{X}}_{2}$	0.0475	1	0.0475	9.22	0.0104	
$\:{\mathrm{X}}_{3}$	0.2517	1	0.2517	48.82	< 0.0001	
$\:{\mathrm{X}}_{4}$	0.1439	1	0.1439	27.91	0.0002	
$\:{\mathrm{X}}_{1}{\mathrm{X}}_{2}$	0.2062	1	0.2062	40.00	< 0.0001	
$\:{\mathrm{X}}_{1}{\mathrm{X}}_{3}$	0.0819	1	0.0819	15.90	0.0018	
$\:{\mathrm{X}}_{1}{\mathrm{X}}_{4}$	9.506E-06	1	9.506E-06	0.0018	0.9665	
$\:{\mathrm{X}}_{2}{\mathrm{X}}_{3}$	0.0084	1	0.0084	1.64	0.2251	
$\:{\mathrm{X}}_{2}{\mathrm{X}}_{4}$	0.0775	1	0.0775	15.04	0.0022	
$\:{\mathrm{X}}_{3}{\mathrm{X}}_{4}$	0.0260	1	0.0260	5.05	0.0442	
$\:{\mathrm{X}}_{1}^{\:\:2}$	0.1994	1	0.1994	38.68	< 0.0001	
$\:{\mathrm{X}}_{2}^{\:\:2}$	0.0062	1	0.0062	1.21	0.2930	
$\:{\mathrm{X}}_{3}^{\:\:\:2}$	0.0892	1	0.0892	17.31	0.0013	
$\:{\mathrm{X}}_{4}^{\:\:\:2}$	0.2182	1	0.2182	42.33	< 0.0001	
Residual	0.0619	12	0.0052			
Lack of Fit	0.0585	10	0.0058	3.45	0.2456	Not significant
Pure Error	0.0034	2	0.0017			
Cor Total	1.73	26				

### Effect of variables on tensile strength

**Fig. 5:**
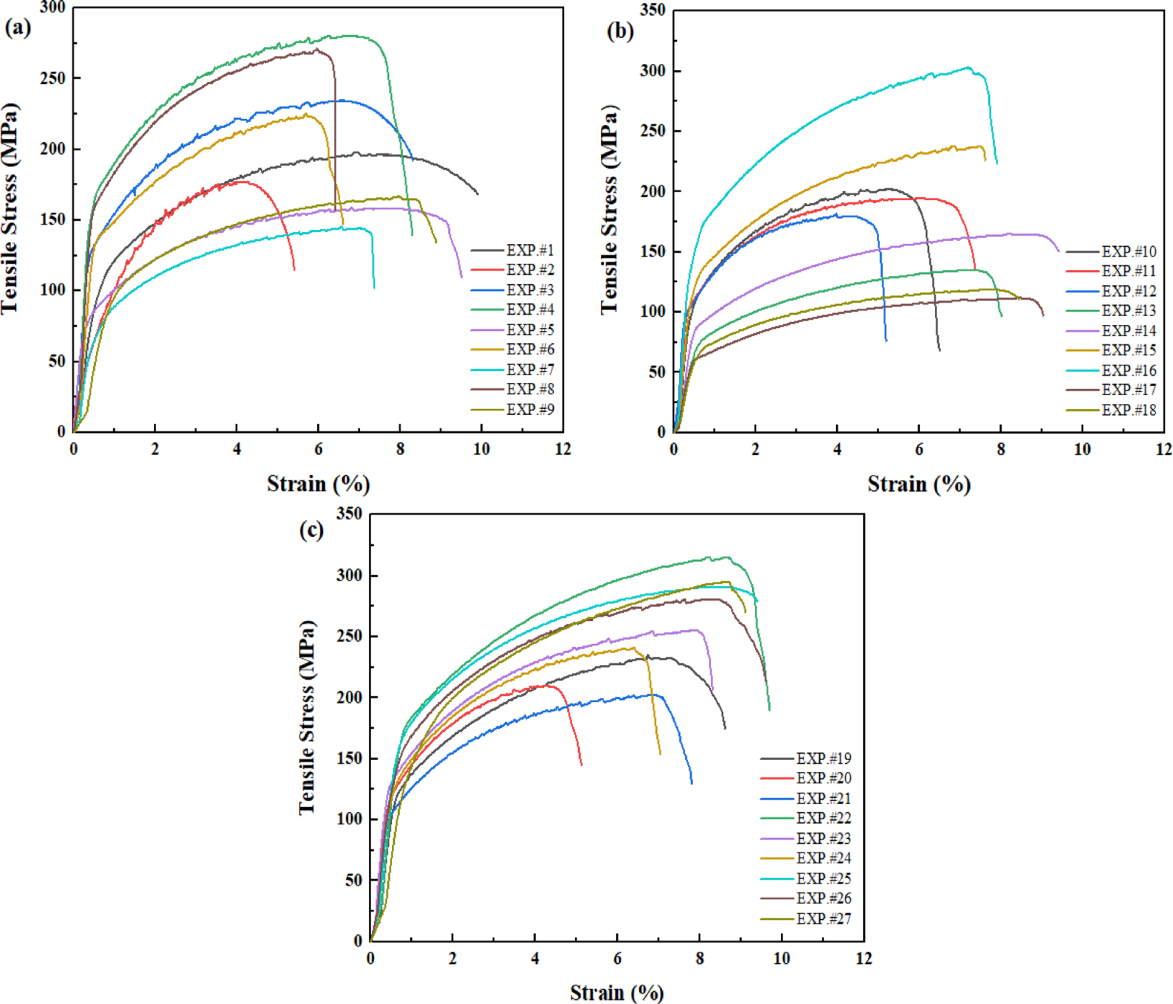
Stress strain diagram for experimental runs 1–9 (**a**), 10–18 (**b**) and 19–27 (**c**).

**Fig. 6:**
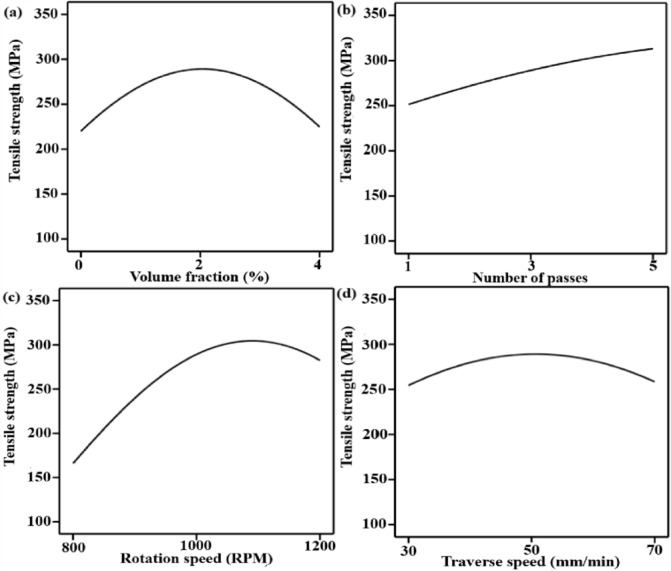
Main effect plots for tensile strength as a function of volume fraction (**a**), number of passes (**b**), rotation speed (**c**) and traverse speed (**d**).

The stress-strain diagram for all experimental run is shown in Fig. 5. The main effect plots show clear, comprehensible trends in tensile strength as influenced by friction stir processing variables as shown in Fig. 6. In Fig. 6a, the volume fraction exhibits a non-linear influence, with tensile strength increasing up to a volume fraction of about 2% before declining at higher values. At low to moderate reinforcement levels, this behavior is explained by improved load transfer and grain refinement, whereas high particle content causes agglomeration, poor interfacial bonding, and stress concentration, which lower tensile strength^67^. The number of passes (Fig. 6b) demonstrates a frequently beneficial effect, which implies that through cumulative severe plastic deformation and dynamic recrystallization, repeated FSP passes enhance microstructural homogeneity, break up particle clusters, and promote progressive grain refinement^68^. Tensile strength increases at an intermediate speed (1000 rpm), and the effect of rotation speed follows a quadratic trend (Fig. 6c). While excessive rotation speed results in overheating, grain coarsening, and matrix softening, which worsens tensile performance, insufficient rotation speed leads to insufficient heat input and poor material flow^69^. Additionally, traverse speed exhibits an ideal response (Fig. 6d), where intermediate speeds produce defect-free microstructures with refined grains by balancing strain rate and heat generation. Higher traverse speeds decrease stirring effectiveness and consolidation quality, while lower traverse speeds cause excessive heat exposure and coarsening^70^.

**Fig. 7:**
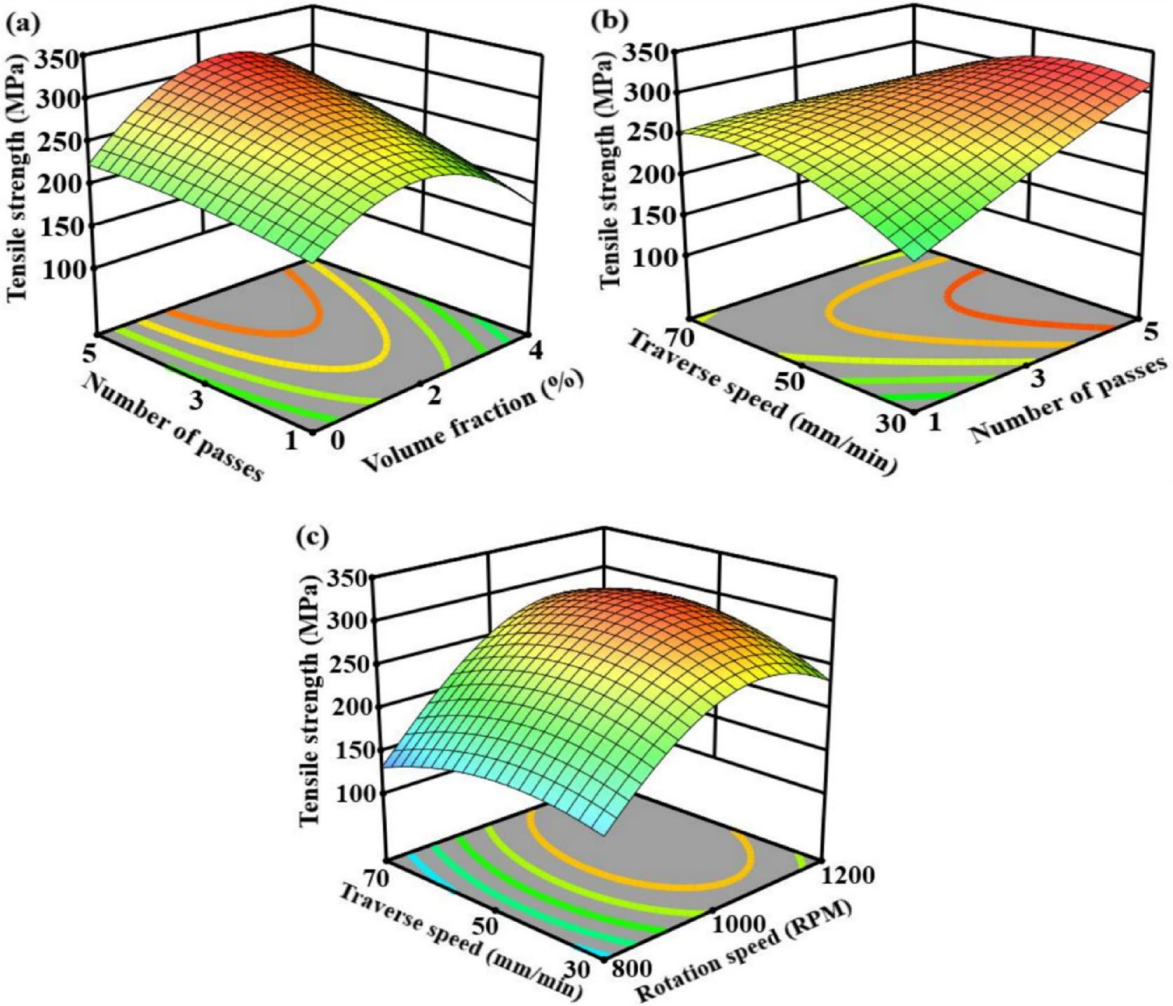
Response surface plot for the tensile strength versus; (**a**) volume fraction and number of passes load, (**b**) number of passes load and traverse speed, (**c**) rotation speed and traverse speed.

The interactive effects of volume fraction and number of passes are depicted in Fig. 7a, where tensile strength increases with both parameters up to a volume fraction of 2% and higher passes. This characteristic is caused by enhanced reinforcement dispersion and synergistic grain refinement because of repeated plastic deformation, whereas an excessive volume fraction causes aggregated particles and local stress concentration even after numerous passes. The interaction between traverse speed and number of passes is exhibited in Fig. 7b, demonstrating that maximum tensile strength is achieved when higher passes are combined with reasonable traverse speed, where it is evident that tensile strength is most influenced by these two variables. Grain coarsening results from excessive heat input at lower traverse speeds, while material flow and consolidation are reduced at high traverse speeds, even with multiple passes^71^. The rotation speed and traverse speed exhibit an explosive quadratic interaction in Fig. 7c, with maximum tensile strength developing at intermediate levels of both variables. This desirable region is associated with a balanced thermomechanical condition that encourages defect-free material flow, fine equiaxed grains, and dynamic recrystallization.

### Effect of variables on yield strength

**Fig. 8:**
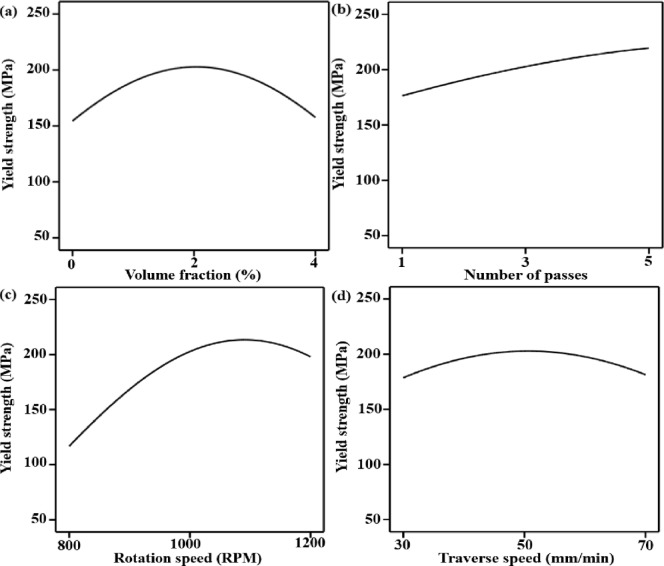
Main effect plots for yield strength as a function of volume fraction (**a**), number of passes (**b**), rotation speed (**c**) and traverse speed (**d**).

The implications of volume fraction (Fig. 8a) exhibit an unambiguous non-linear trend, with yield strength improving up to a volume fraction of about 2% and decreasing at higher volume fractions. Initially, as the WC volume fraction increases, the composite’s yield strength increases. The higher work hardening rate of the materials containing particles contributes to the increase in yield strength. Additionally, the strength of the composites is impacted by the uniform distribution of WC particles. The yield strength decreases as the WC volume fraction rises further. Particle agglomeration, increased defects, and microporosity in the composite at higher WC volume fraction could be the cause of this^72^. Yield strength increases monotonically with the number of passes (Fig. 8b), demonstrating the cumulative effect of repeated severe plastic deformation. Several passes increase grain boundary density and dislocation barriers, which directly increase yield strength, by intensifying dynamic recrystallization, refining grain size, and homogenizing reinforcement distribution. Yield strength is influenced by rotation speed in a parabolic manner (Fig. 8c), reaching maximum values at intermediate rotational speeds (1000 rpm). Insufficient heat input limits plastic deformation and material flow at slower speeds, which limits recrystallization and produces relatively coarse grains. On the other hand, increased thermal input from excessive rotation speed results in recovery-dominated softening, grain coarsening, and a partial loss of strain hardening capacity, all of which lower yield strength^73^. Additionally, traverse speed (Fig. 8d) exhibits an ideal response, with yield strength reaching its maximum at moderate travel rates (50 mm/min). High traverse speeds result in incomplete consolidation and heterogeneous deformation zones, whereas low traverse speeds increase grain growth and dynamic recovery by extending thermal exposure. By eroding microstructural barriers to dislocation motion, both extremes reduce yield strength^74^.

**Fig. 9:**
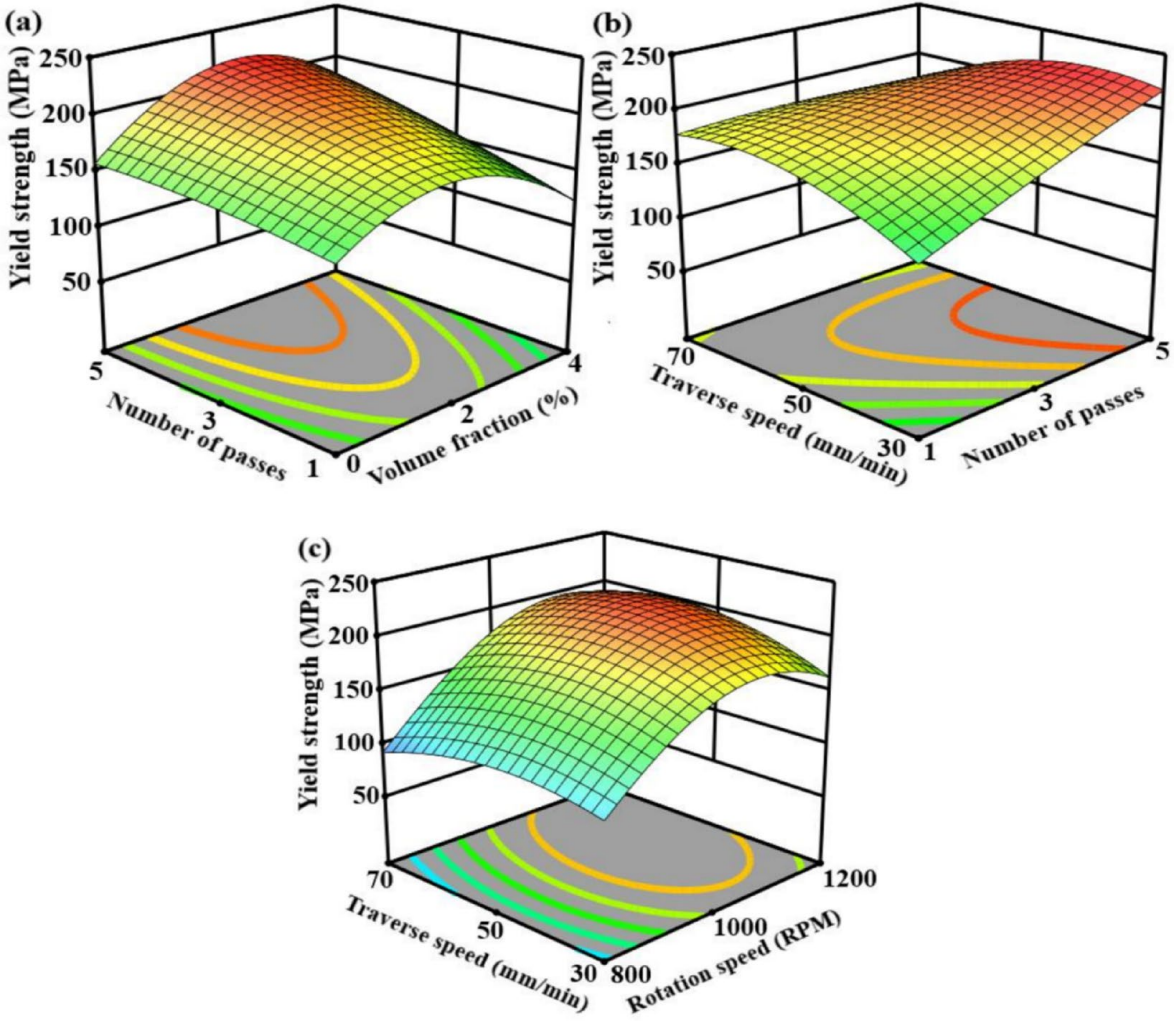
Response surface plot for the yield strength versus; (**a**) volume fraction and number of passes load, (**b**) number of passes load and traverse speed, (**c**) rotation speed and traverse speed.

The interaction between volume fraction and number of passes is depicted in Fig. 9a, where yield strength rises with both parameters up to an ideal region at intermediate reinforcement content and higher passes. enhanced dislocation density, synergistic grain refinement, and efficient load transfer produced by uniform reinforcement dispersion under repeated severe plastic deformation contribute to this enhancement. Particle agglomeration and interfacial defects cause yield strength to drop beyond the ideal volume fraction even with more passes^75^. The interaction of traverse speed and number of passes is shown in Fig. 9b, showing that higher passes combined with moderate traverse speeds yield the highest yield strength. High traverse speeds decrease plastic flow and consolidation, while low traverse speeds encourage excessive heat exposure and grain coarsening, both of which result in decreased yield strength even under multi-pass conditions. The quadratic interaction between rotation speed and traverse speed is shown in Fig. 9c, with highest yield strength developing at intermediate levels of both variables. Fine equiaxed grains and a high dislocation density are stabilized in this area, which corresponds to an ideal balance between strain rate and thermal input^76^.

### Effect of variables on elongation

**Fig. 10:**
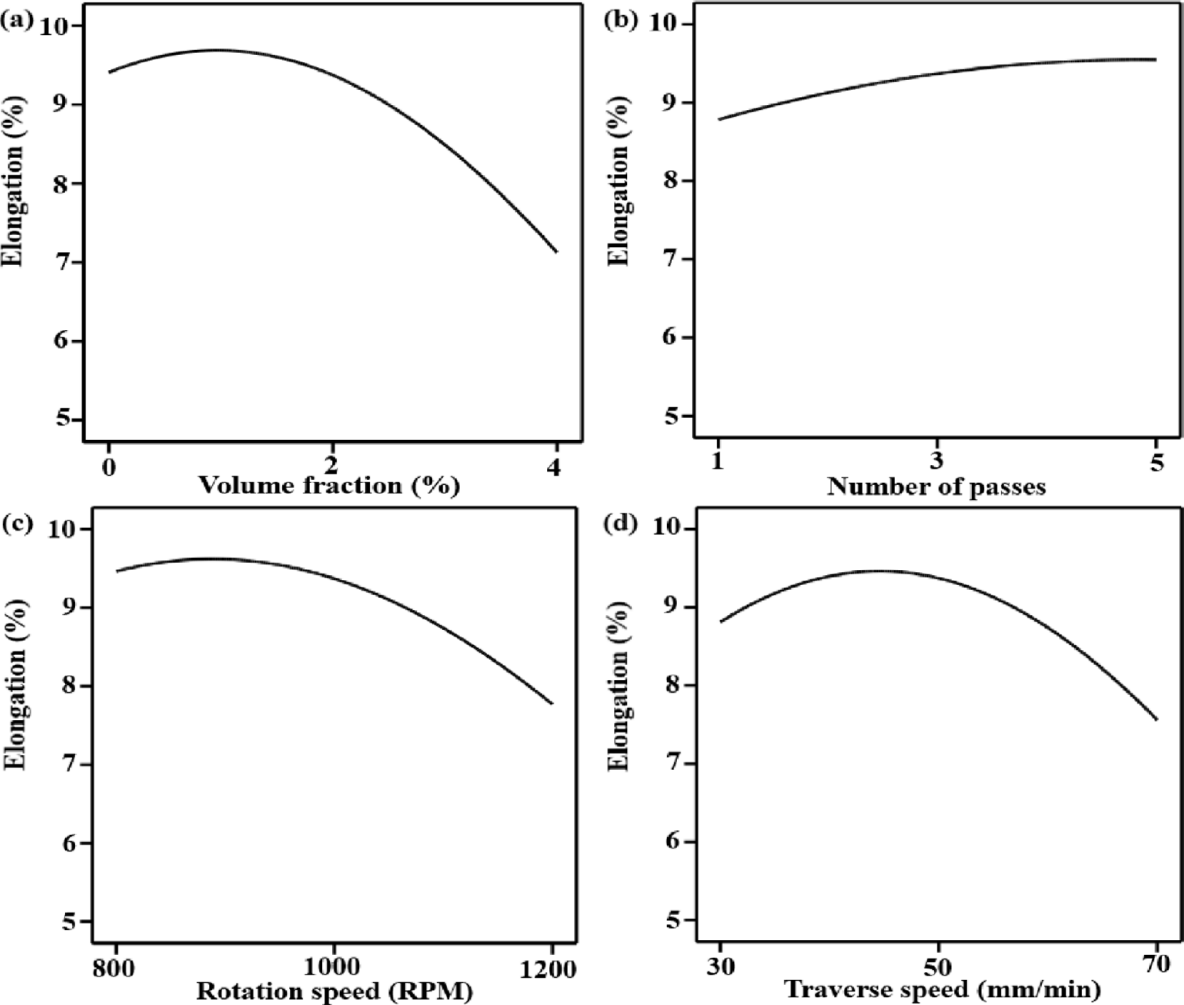
Main effect plots for elongation as a function of volume fraction (**a**), number of passes (**b**), rotation speed (**c**) and traverse speed (**d**).

The volume fraction impact (Fig. 10a) demonstrates a non-linear trend, with elongation slightly increasing at low reinforcement content and then significantly decreasing at higher volume fractions. Particle aggregation weakened interfacial bonding, and elevated stress concentration limit dislocation motion and strain compatibility as volume fraction rises above the ideal level, resulting in early fracture and lowered elongation^77^. The number of passes (Fig. 10b) shows an increase in elongation, implying that ductility is effectively enhanced through numerous FSP passes. A more uniform strain field during tensile loading results from multiple passes that improve material flow, remove micro-voids and tunnel defects, produce fine equiaxed grains through progressive dynamic recrystallization, and homogenize reinforcement distribution. This enhances strain accommodation without localized damage, increasing elongation^78^. The interaction between rotation speed and elongation appears in Fig. 10c, with the highest ductility taking place at intermediate speeds. Insufficient heat input restricts plasticization and recrystallization at low rotation speeds, resulting in decreased elongation. Elongation peaks at moderate travel rates, and traverse speed (Fig. 10d) additionally shows optimal behavior. Low traverse speeds cause microstructural coarsening and excessive heat accumulation, while high traverse speeds shorten interaction times and decrease stirring effectiveness. These extremes limit uniform plastic deformation and decrease elongation^79^.

**Fig. 11:**
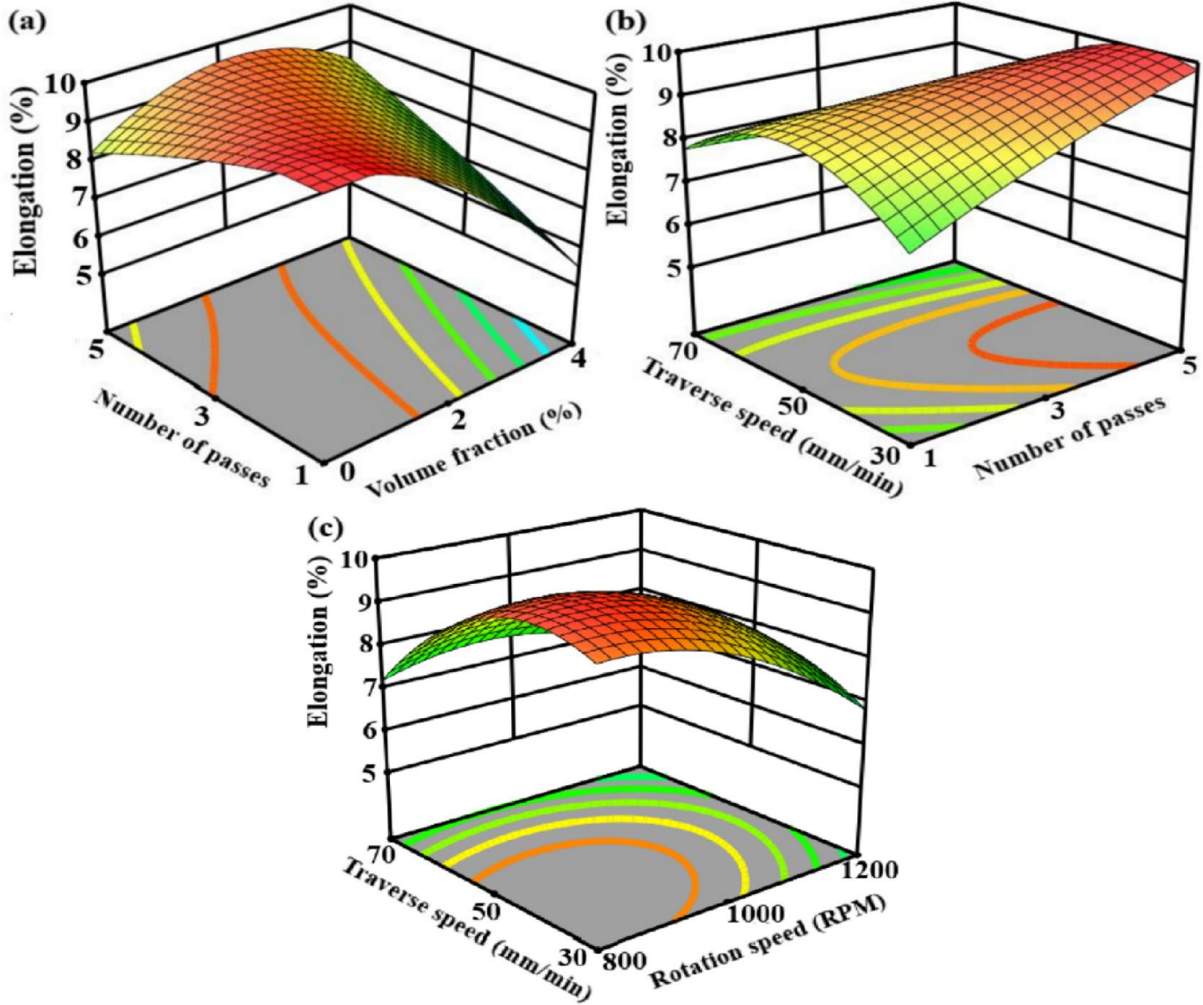
Response surface plot for the elongation versus; (**a**) volume fraction and number of passes load, (**b**) number of passes load and traverse speed, (**c**) rotation speed and traverse speed.

Response surface plots in Fig. 11 show the combined implications of important friction stir processing variables on elongation. As illustrated by Fig. 11a, elongation increases with the number of passes at low to intermediate reinforcement volume fractions for the reason of improved particle distribution, improved material flow, and grain refinement; at higher volume fractions, however, there is a noticeable decline, which is explained by increased strain localization and particle aggregation that decreases ductility. Figure 11b indicates that elongation increases with traverse speed and number of passes up to an ideal region. Subsequently, microstructural coarsening and decreases in ductility are brought on by excessive heat input at low traverse speeds. The highest elongation is achieved at intermediate levels of both parameters, where adequate heat generation and stable plastic flow promote dynamic recrystallization without causing grain growth, as shown by the interaction caused by rotation speed and traverse speed in Fig. 11c. The results presented here indicate that a compromise between thermal input, strain rate, and reinforcement dispersion controls ductility in friction stir processed aluminum matrix composites^80–83^.

### Tensile performance optimization

Identifying a desired point in the design space is the aim of response surface optimization research. Based on design expert software, the optimization criterion can produce many possible solutions. Establishing a minimum, maximum, or specific area where the response is stable may be required. The input variables are optimized by models created for the maximum values of elongation, yield strength, and tensile strength. The optimized values of variables were found to be a volume fraction of (2%), number of passes (5), tool rotation speed(1000 rpm), and traverse speed (30 mm/min) to get the maximum tensile strength (315 MPa), yield strength (221 MPa) and elongation (9.7%).

### Microstructural evolution

**Fig. 12:**
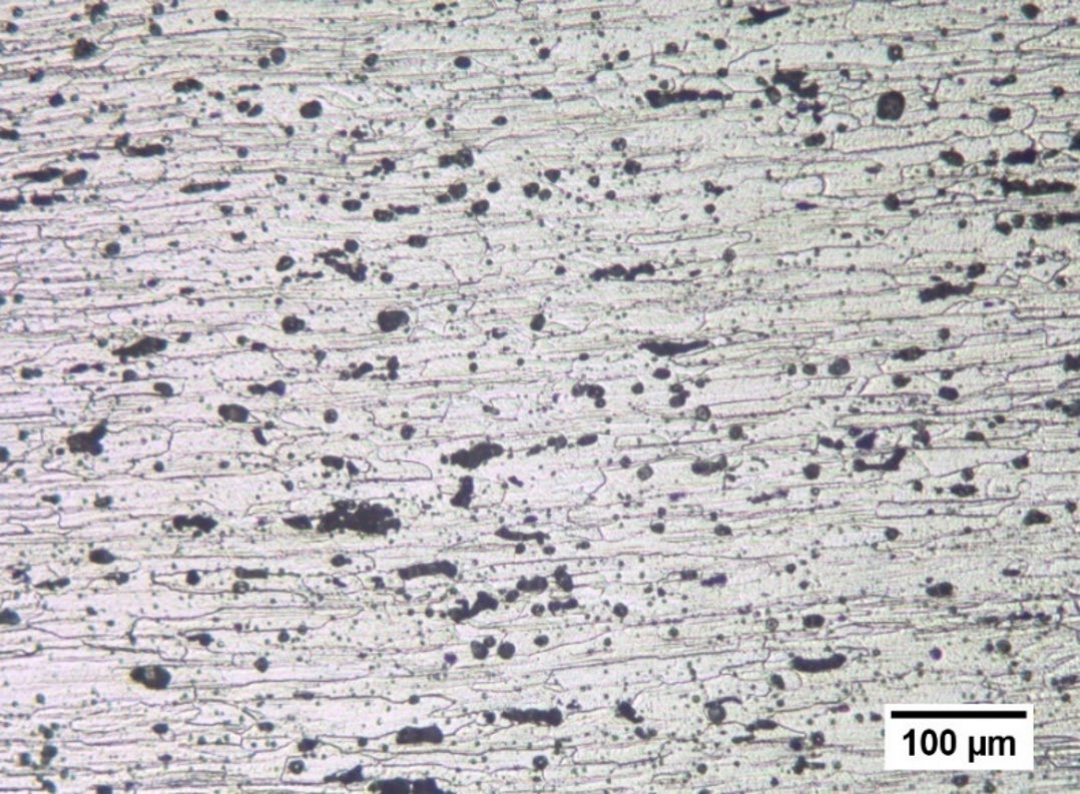
Optical microstructure for base metal (AA6061-T6).

**Fig. 13:**
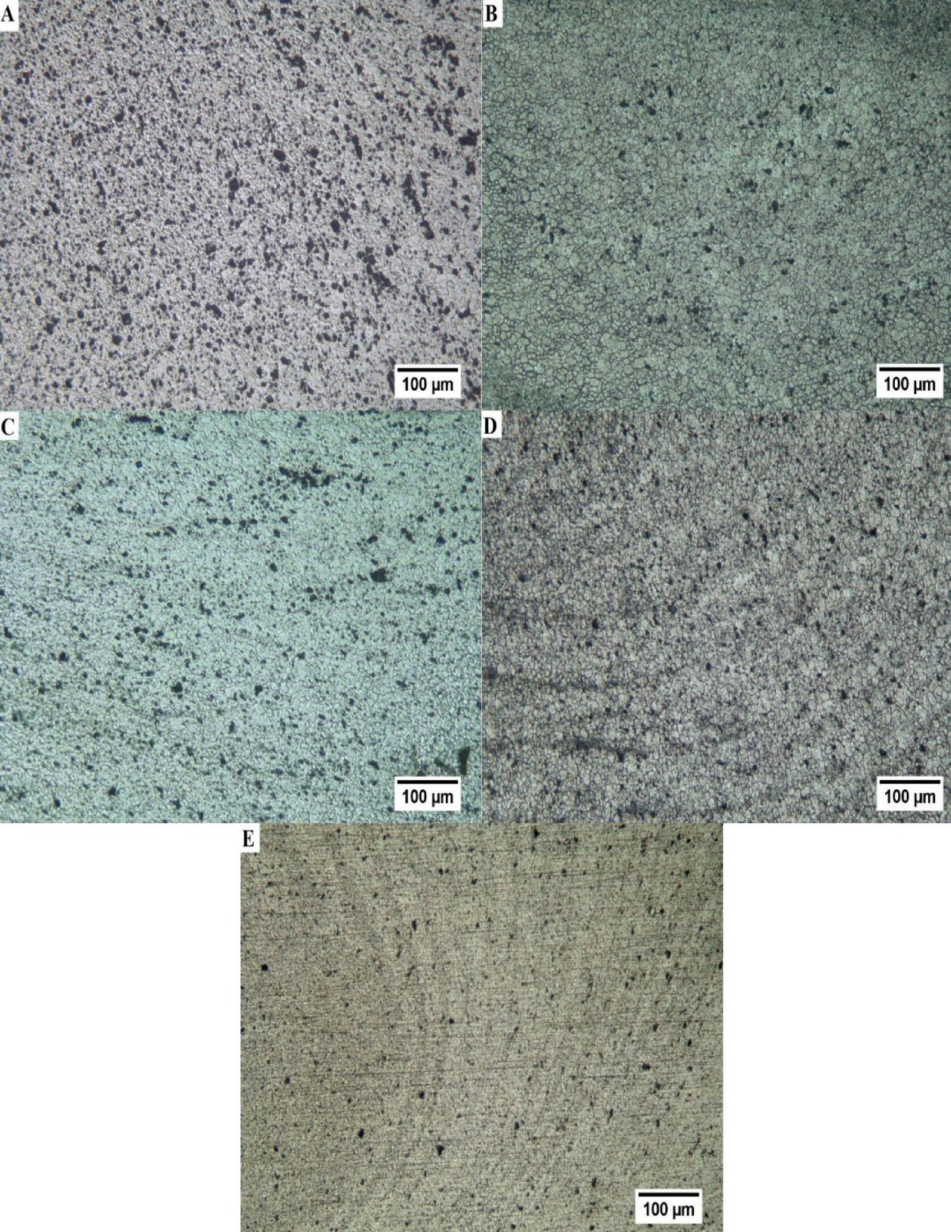
Optical microscopic images of the stir zone at (**a**) EXP.3; (**b**) EXP.8; (**c**) EXP.16; (**d**) EXP.20; and (**e**) EXP.22.

The microstructure of the base alloy (AA6061-T6) is shown in Fig. 12. The microstructure demonstrates the impact of previous thermomechanical processing with elongated grains oriented parallel to the rolling direction. The aluminum matrix possesses a variety of intermetallic phases that are heterogeneously distributed and show up as dark particles that are found both inside and along the grain boundaries. A comprehensive microstructural analysis of the stir zone in friction stir processed (FSP) Al6061-T6/WC composites under different values of traverse speed, rotation speed, number of passes, and reinforcement volume fraction is displayed in Fig. 13. High strain and the possibility of dynamic recrystallization (DRX) affect the stir zone in FSP. The weld nugget’s recrystallized microstructure is made up of well-stirred, harmonious grains. In FSP, the stretched microstructure of the parent metal produces dynamically recrystallized (DRX) equiaxed grains. This suggests that severe plastic deformation in FSP creates low angle misoriented grain boundaries and splits the original grains, creating a large number of nucleating zones for recrystallization^84,85^. The stir zone shows a refined and equiaxed grain structure compared with the base material in the unreinforced condition (Fig. 13a, EXP. 3), processed at zero WC content with 5 passes, 1000 rpm, and 50 mm/min. This confirms the dominance of severe plastic deformation and dynamic recrystallization inherent to FSP of Al6061-T6^86^.

A significant change in microstructural characteristics can be observed with incorporation of WC particles at 2 vol% (Fig. 13b and c). The microstructure exhibits slightly refined grains with observable particle clusters at lower passes (3 passes) and higher processing speeds 1200 rpm and 70 mm/min (Fig. 13b, EXP. 8), implying insufficient cumulative strain and little residence time for efficient particle breakup and dispersion. On the other hand, a substantially more uniform dispersion of WC particles within the aluminum matrix, accompanied by finer and more equiaxed grains, is achieved by increasing the number of passes to 5, 1200 rpm, and 50 mm/min. (Fig. 13c, EXP.16). Recurring stirring cycles are responsible for this improvement, since they increase particle fragmentation, break up agglomerates, and encourage consistent mixing through a series of recrystallizations^87^.

The stir zone in Fig. 13d, (EXP.20) which corresponds to the higher WC content of 4 vol% processed with 3 passes at 1200 rpm and 50 mm/min, exhibits a distinct onion-ring morphology. Recurring material deposition during tool rotation causes these concentric flow bands, which are exacerbated by the higher WC particle concentration’s increased resistance to plastic flow. Particle-rich and particle-lean layers alternate because of the high density of hard WC particles’ promotion of localized strain accumulation and cyclic variation in material velocity. These onion-ring structures, which are frequently attributed to particle agglomeration, uneven heat distribution, and non-uniform recrystallization, demonstrate incomplete homogenization and may serve as preferred locations for stress localization under mechanical loading^86,87^. Onion-ring features are considerably more diffuse and refined in Fig. 13e, (EXP.22) which was processed at 2 vol% WC with five passes, 1000 rpm, and a lower traverse speed of 30 mm/min. The rings are uniformly distributed and less pronounced, indicating improved material flow and the gradual disintegration of WC aggregates over subsequent passes. While a higher number of passes encourages repetitive shear deformation, which produces smoother flow bands and better particle distribution, the lower traverse speed increases heat input and material residence time. The onion-ring pattern in this situation indicates a well-developed stir zone with enhanced microstructural uniformity and interfacial bonding, as opposed to flow instability, which is represented by stable and controlled plastic flow^90^. Grain refinement and dislocation strengthening were the two primary factors that had a major impact on the mechanical properties of the material in the stirred zone. By adding WC particles, the tensile performances were greatly increased due to particle homogeneity, grain size refinement, and dislocation pinning effects^91,92^.

**Fig. 14:**
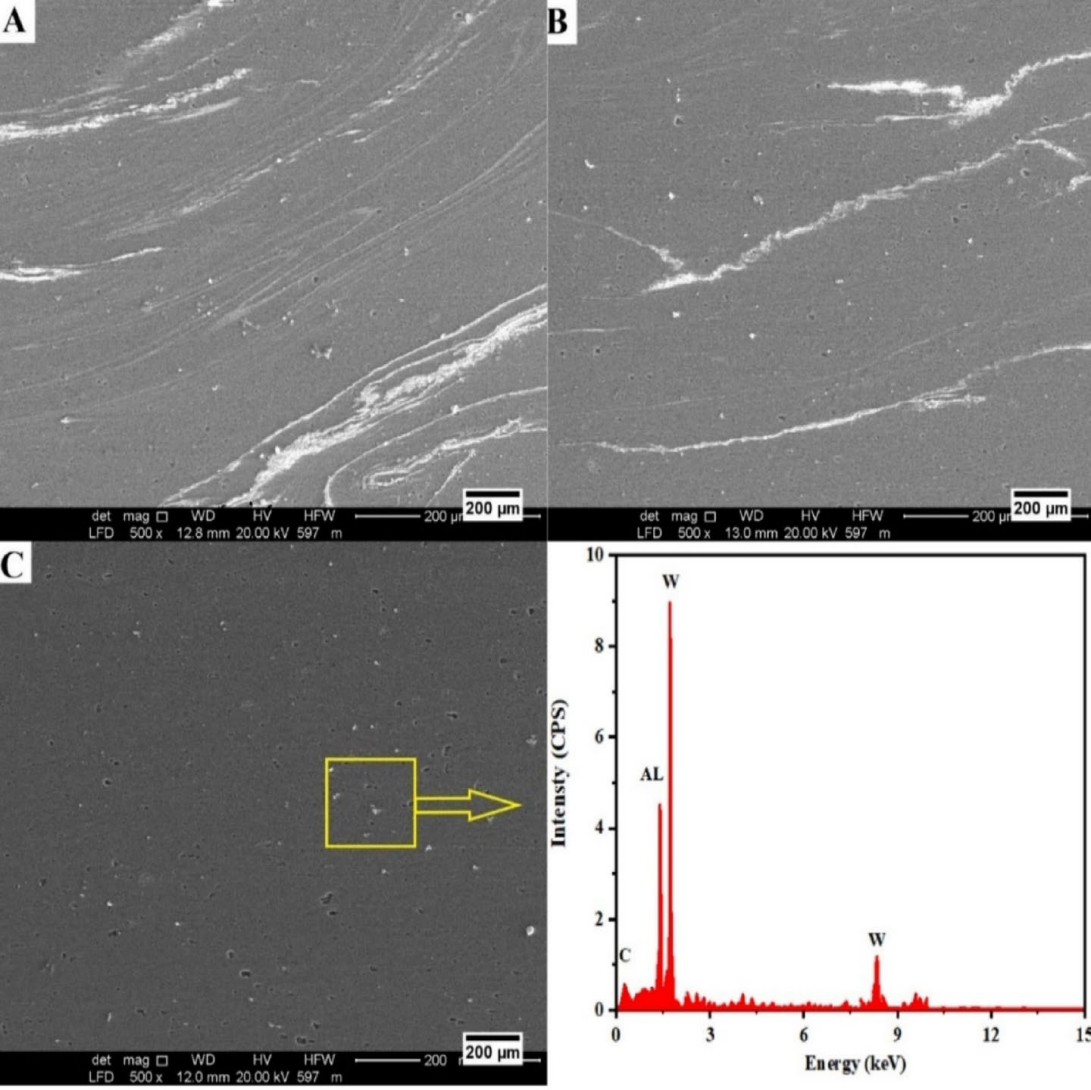
SEM images of the stir zone at (**a**) EXP.18; (**b**) EXP.20; EXP.22 and corresponding EDX (A6061-T6/WC).

SEM images of the friction stir processing (FSP) of the AA6061-T6/WC surface composites in the stir zone are shown in Fig. 14. The observation of partially fragmented features and elongated material flow bands in Fig. 14a suggests insufficient plastic deformation and disparate reinforcement dispersion at rotation speed of 800 rpm^93^. Raising the tool rotation speed to 1200 rpm in Fig. 14b while keeping the volume fraction and number of passes constant improves plastic flow and the dispersion of WC particles, but localized shear bands brought on by severe thermo-mechanical deformation are still visible^94^. The agglomerated reinforcement particles are broken up and evenly distributed throughout the stir zone by the constant stirring action and the matrix’s plastic flow. Figure 14c shows a more uniform and refined microstructure, which is explained by the interaction of a traverse speed (30 mm/min), 5 passes, and WC volume fraction (2%), which encourages adequate heat generation and repeated material mixing^95^. Finer grains and strengthening precipitates, a more uniform distribution of reinforcement particles, and stronger particle matrix interfacial bonding are all responsible for this improvement in the WC-reinforced composite. The provided SEM micrographs show how well surface composites are produced, with a uniform distribution of reinforcements. The presence of aluminum as the matrix element is confirmed by the corresponding EDX spectrum, which shows prominent peaks for Al, W, and C. This suggests that there was no detectable contamination or undesirable reaction products during the successful incorporation of the WC reinforcement.

### Evaluation of fracture morphology

**Fig. 15:**
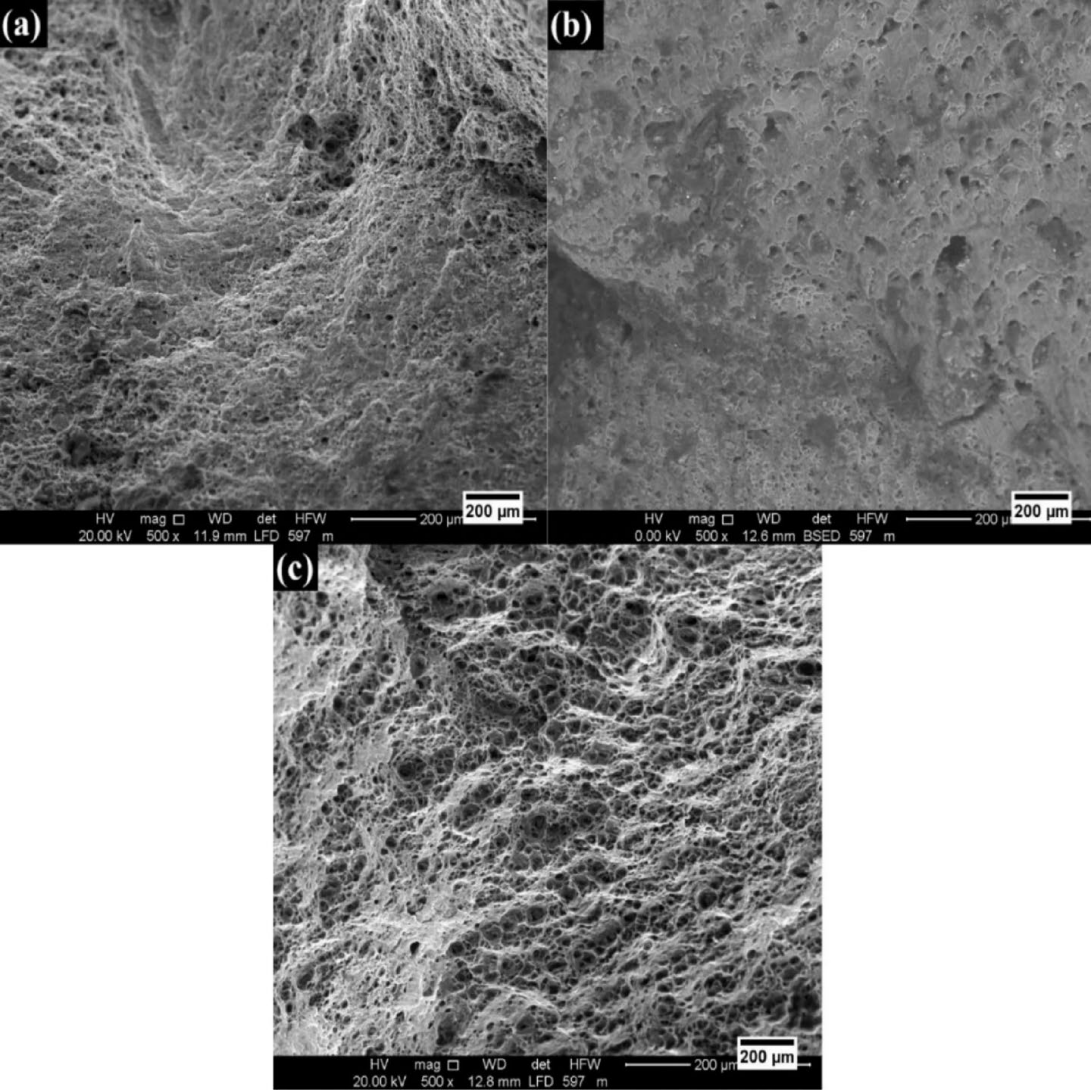
fracture surface images of the stir zone at (**a**) EXP.18); (**b**) EXP.20; and (**c**) EXP.22.

Figure 15 shows the fractured surface morphologies of the stir zone, which disclose the fracture mechanisms of AA6061-T6/WC surface composites under various friction stir processing parameters. Shallow dimples scattered with tear ridges and cleavage planes dominate the fracture surface in Fig. 15a, signifying the mode of brittle and ductile fracture. This indicates limited plastic deformation and early crack initiation brought on by inadequate heat input and an uneven distribution of WC particles at rotation speed of 800 rpm. Greater rotational speed enhances plastic flow, but excessive heat and strain localization can encourage interfacial decohesion and micro-void coalescence around hard WC particles, as shown in Fig. 15b, a mixed-mode fracture with tiny dimples and confined brittle regions. A ductile fracture mechanism caused by refined grains, homogeneous particle dispersion, and efficient load transfer between the matrix and reinforcement is typified by the uniformly dimpled morphology with fine and deep equiaxed dimples shown in Fig. 15c^96^. The strength-ductility balance of aluminum matrix surface composites is determined by the fracture behavior, which is directly correlated with FSP. Interfacial bonding between the Al matrix and WC nanoparticle reinforcements was accomplished, as is also accomplished elsewhere^97^.

## Conclusion

In this study, response surface methodology (RSM) using design expert software was used to analyze the tensile strength, yield strength, and percentage elongation at the nugget zone of FSP AA6061/WC surface nanocomposites. The results contribute to the establishment of the following conclusion:

1. The FSP procedure was successfully used to create aluminum matrix composites with WC nanoparticles and AA 6061-T6 alloy as the matrix.
2. Empirical correlations were developed to predict the output responses of FSP AA6061/WC surface nanocomposites, including tensile strength, yield strength, and % elongation.
3. The tensile strengths of the hybrid aluminum matrix composites reinforced with WC nanoparticles significantly improved by increasing the FSP passes. Grain refinement, DRX, and an increase in dislocation density are the primary causes of this improvement.
4. The optimum surface nanocomposite with a maximum tensile strength (315 MPa), yield strength (221 MPa), and elongation (9.7%) was created at volume fraction 2%, number of passes 5, rotation speed 1000 rpm, and traverse speed 30 mm/min.
5. The AA6061-T6 matrix’s stir zone had uniform distribution of the reinforced WC nanoparticles. The diffusion of WC nanoparticles in the AA6061-T6 matrix was found to have a clear interface between the reinforcement particles and matrix, with no reaction inhibition.
6. The results of the analysis of variance revealed that traverse speed had little effect on tensile properties, while tool rotation speed was the most significant process variable.
7. The development of metal matrix composites with WC nanoparticles inclusions using FSP has attracted attention due to the growing need for lightweight and high-performance materials in military, automotive, and aerospace applications, such as defensive armor.

## Data Availability

All data generated or analyzed during this study are included in this published article.
